# New Experimental Evidence for Drying Shrinkage of Alkali-Activated Slag with Sodium Hydroxide

**DOI:** 10.3390/ma16165659

**Published:** 2023-08-17

**Authors:** Marco Sirotti, Brice Delsaute, Stéphanie Staquet

**Affiliations:** BATir Department, Université Libre de Bruxelles, CP194/02, 50 Avenue F.D. Roosevelt, 1050 Brussels, Belgium; brice.delsaute@ulb.be (B.D.); stephanie.staquet@ulb.be (S.S.)

**Keywords:** drying shrinkage, autogenous shrinkage, alkali-activated materials, creep, microcracking, carbonation

## Abstract

Alkali-activated slag (AAS) is emerging as a possible and more sustainable alternative to Ordinary Portland Cement (OPC) in the construction industry, thanks to its good mechanical and chemical properties. Conversely, the effects of its high drying shrinkage are still a concern for its long-term durability. This study aims to investigate the drying shrinkage behaviour of six AAS/sodium hydroxide mortar compositions and the main phenomena affecting their drying shrinkage behaviour. Specifically, the molarity, solution-to-binder ratio (s/b), autogenous shrinkage, creep compliance, microcracking, and carbonation are considered as possible causes of the differences between AAS and OPC. The results show that it is not possible to correlate the shrinkage magnitude with the molarity of the activating solution, while an increase in the s/b increases the drying shrinkage. Concerning the other factors, autogenous deformation remains significant even after a period of 112 days, while the creep compliance is definitely affected by the drying process but does not seem to affect the shrinkage magnitude. Furthermore, the presence of microcracks caused by the drying process definitely influences the drying shrinkage. Finally, carbonation depends on the molarity of the activating solution, even though its effects on the material are still unclear.

## 1. Introduction

As the production of Ordinary Portland Cement inherently releases high quantities of CO_2_ into the atmosphere [1,2,3,4,5], it is crucial to investigate possible alternatives to cementitious materials that would reduce the carbon footprint of the construction industry. Alkali-activated slag combines an eco-friendly performance [6,7,8] with promising mechanical properties and chemical durability [5,6,9]. However, its drying shrinkage seems to be much higher than that observed for OPC, raising concerns for its long-term durability [6,10,11,12].

The numerous studies available on OPC allow us to understand the mechanisms behind drying shrinkage and how the drying conditions, composition, and curing time affect the volume stability. Nonetheless, this is not true for alkali-activated slag; the available studies on the topic normally investigate the effects of changing either the drying conditions or the composition. If we know that reducing the external relative humidity does not necessarily increase the shrinkage [6,13,14,15], as occurs for Portland cement, there are several possibilities as to why said behaviour is observed. In some cases, a higher creep deformation or relaxation at certain relative humidity levels has been observed [6,13], which may explain the difference in behaviour between OPC and AAS. Other studies show a strong formation of microcracks and carbonation of the material [16,17]; both phenomena are known to affect the shrinkage magnitude of a porous material. To the best knowledge of the authors, none of the studies investigated shrinkage, creep, microcracking, and carbonation of alkali-activated slag in different drying conditions at the same time in order to shed light on the relationship between the different properties and the effects they have on one another.

On the topic of the composition, some authors investigated the effect of the activator on the drying shrinkage and found that the use of sodium silicate causes a refinement in the pore structure [18,19,20] and a subsequent increase in the drying shrinkage [12,21] when compared to other activators, such as sodium hydroxide or sodium carbonate. In addition, increasing the content of Na_2_O also increases the drying shrinkage, regardless of the activator [11,12,21,22,23]. On the other hand, said studies were carried out at specific relative humidity levels and do not always present a parametric change in the mix composition. Therefore, it is still not clear how changing the molarity or the activator quantity affects the volume stability of slag when exposed to different RH levels. Finally, it is known that autogenous deformation is a significant part of the total shrinkage of alkali-activated slag at early age [11,24], but it is still unclear how alkali-activated slag behaves in purely drying conditions, that is to say when autogenous deformation can be considered negligible.

The present study investigates the drying shrinkage of alkali-activated slag in different relative humidity conditions compared to Ordinary Portland Cement. Specifically, the results highlight the difference in behaviour between AAS and OPC but also provide new experimental evidence on the drying shrinkage of AAS considering the changes in molarity of the activating solution and in the solution-to-binder ratio. In addition, in order to better explain the drying shrinkage results, autogenous shrinkage, creep compliance, E-modulus, flexural and compressive strength, and carbonation are tested as well. Specifically, the study consists of three different experimental campaigns that will be introduced more extensively in the following sections. The experimental campaigns are:

- A study on the drying shrinkage in different relative humidity (RH) conditions and the autogenous shrinkage for different AAS compositions in comparison with OPC;
- A study on the creep compliance for one of the compositions with different curing histories;
- A study on the E-modulus, flexural strength, compressive strength, and carbonation for one of the compositions.

## 2. Drying and Autogenous Deformation and Mass Variation

### 2.1. Mortar Composition

The present study investigates six different alkali-activated slag compositions. In all cases, the chosen precursor is ground granulated blast furnace slag with the oxide composition shown in Table 1. The study also investigates OPC with a water-to-cement ratio of 0.5, which is used as a reference. Specifically, the chosen Portland cement is a CEM I 52.5 N cement with a minimum of 95% clinker.

The chosen activators are sodium hydroxide solutions with different molarities of 0.5, 2, and 8 M; the reason for such a choice is the possibility to investigate the effect of the concentration on the shrinkage of the material. In addition, the solution-to-binder ratio is set to 0.5 and 0.8 to study the effect of water and solution content on AAS. Finally, to reduce the formation of microcracks, the tests are carried out on the mortar scale; the sand-to-paste ratio is kept constant at 1, as preliminary studies proved it is impossible to obtain a proper mix with a higher sand content, especially for high molarity compositions. Before casting, the sand was oven-dried at 105 °C for 24 h and stored in airtight containers to ensure a constant degree of saturation. A summary of the mix compositions is reported in Table 2.

### 2.2. Experimental Procedure

#### 2.2.1. Drying Deformation

The samples used to record the drying deformation were prisms prepared according to ASTM C490 [26], with a square section of 25 mm and a length of 285 mm. After casting, the top part of the samples was covered with a plastic film to ensure sealed conditions and they were kept in a climatic chamber at 20 °C and 70% RH; the initial curing time varied between 48 and 72 h, according to the composition’s setting time. Afterwards, the specimens were unmoulded and wrapped in a plastic sheet until the total sealed curing period was 28 days, always at 20 °C. It was assumed that the long curing period is able to minimise the effects of self-dessication on the shrinkage measurement. After the curing period was over, the extremities of the samples were covered in aluminium foil, prepared for measurement, measured to know their initial position, weighed, and finally exposed to the target RH. To ensure a good repeatability, three samples per composition and per relative humidity level were cast.

The test took place at a constant temperature of 20 °C and at different levels of relative humidity, namely 30, 55, and 75%; the change in external relative humidity allows us to investigate the effects of the drying conditions with a significant difference in the RH levels. To ensure the proper RH, the samples were stored in sealed boxes over a saturated salt solution; the salts used to obtain the target RH are shown in Table 3. The chosen salts do not ensure the exact target relative humidity but still allow us to have values different enough from one another to appreciate the difference in RH between the different boxes. To ensure the absence of any changes in drying conditions, the relative humidity and temperature were checked before every measurement using an ALMEMO FHA 646 R device (Ahlborn Mess- und Regelungstechnik GmbH, Holzkirchen, Germany) with an accuracy of ±2% RH at 25 ±3 °C and ±0.2 °C between 0 and 70 °C.

**Figure 1:**
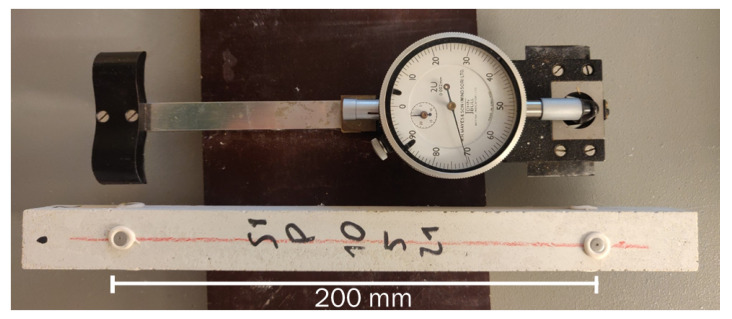
Drying shrinkage sample and measuring device.

The measurement of drying shrinkage was performed using a mechanical strain gauge (DEMEC) (Mayes Group, Windsor, UK) with a gauge length of 200 mm in order to avoid extremity effects. The DEMEC system directly measures the strain of the sample in comparison to an invar reference bar, with a resolution of 8 microstrains (με). The device measures how the distance between two specific points on each side of the sample changes over time. Specifically, said measurement points are materialised by gluing thin metal discs with a blind hole, adapted to accurately accommodate the measuring part of the instrument. The materialisation of the measurement points on the sample and the DEMEC device is shown in Figure 1.

The main disadvantage of measuring the drying deformation with the DEMEC device is the impossibility of continuous monitoring over time. Still, preliminary studies showed a high loss of accuracy when using continuous displacement sensors due to the necessity of weighting the mass change over time on the very same samples for which the shrinkage is recorded; regardless of the use of a stainless steel reference to verify the stability of the sensor positioning on the test rig, a discrepancy was recorded whenever the samples were weighted. As a conclusion, the authors opted for a discontinuous measurement that guarantees the stability of the readings over time even though it does not allow for a frequent measurement rate, especially at the beginning of the test.

#### 2.2.2. Autogenous Deformation

The autogenous deformation was measured on samples with the same size and characteristics as the ones used for the measurement of the drying deformation. Similarly, the instrument used was a DEMEC with a gauge length of 200 mm. The test started right after demoulding the samples after 24–74 h according to the setting time of the composition, but only the results obtained after 28 days were taken into account in order to compare the results with what was observed in drying conditions. In order to ensure a proper sealing of the samples, they were covered with a double layer of self-sealing aluminium foil and weighed regularly.

#### 2.2.3. Mass Variation

The mass variation of AAS was measured on the same samples used for the drying shrinkage. Specifically, the weight change of the samples was recorded using a balance with a resolution of 0.1 g, corresponding to 0.02–0.03% of the initial weight of the samples.

### 2.3. Results and Discussion

#### 2.3.1. Drying Deformation

**Figure 2:**
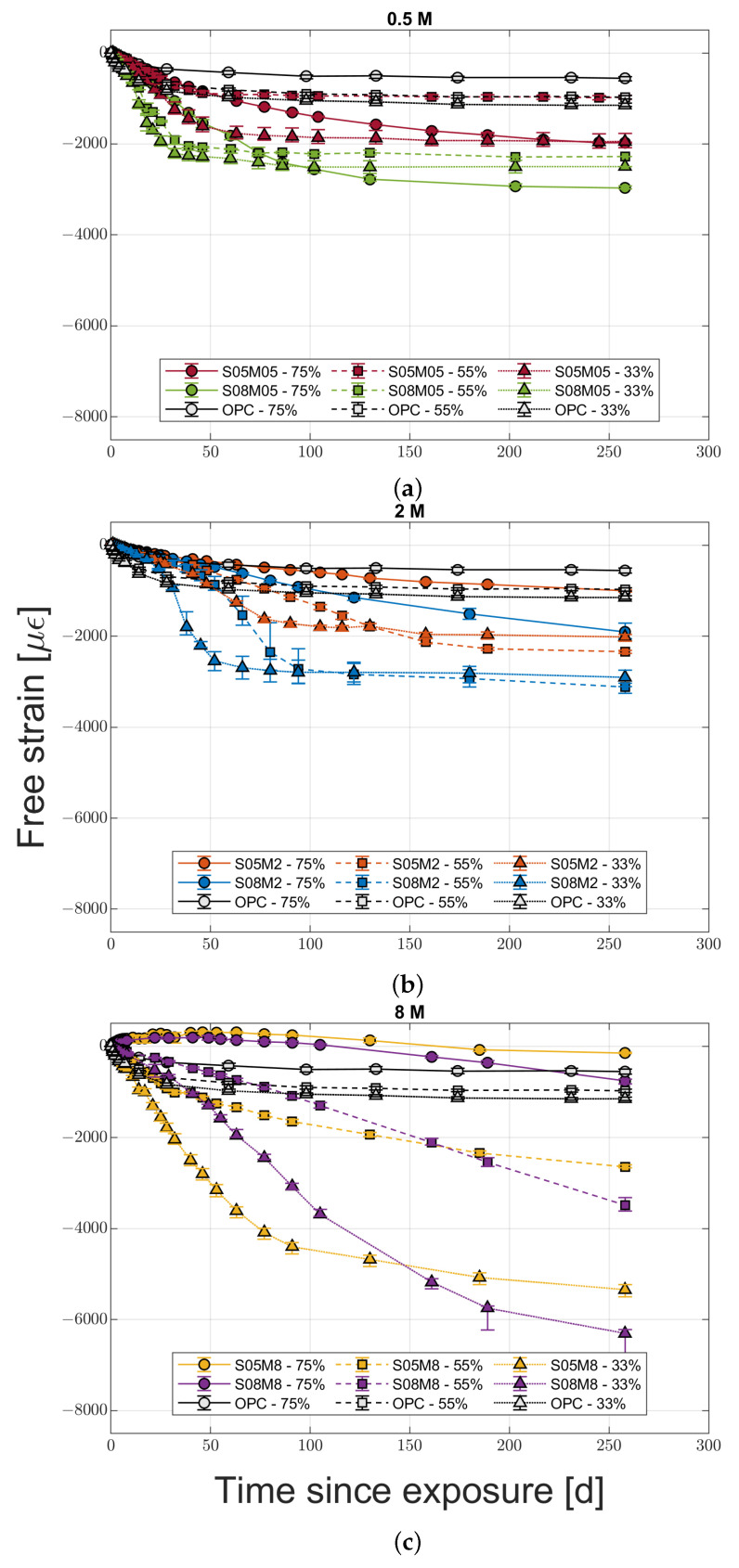
Drying shrinkage evolution for the 0.5 M (**a**), 2 M (**b**), and 8 M (**c**) compositions compared to OPC.

The drying deformation evolutions for the six compositions and OPC are shown in Figure 2a–c. For all the mortars, the last measurement was taken at 258 days after the exposure to drying. In all cases, the mass change was less than 0.15% over 30 days. The values represented in the graphs are the average for the three samples tested, which in turn are the average of the four different sides of each sample, while the error bars represent the relative minimum and maximum values recorded between the different samples.

From a general perspective, it is possible to notice how OPC presents a lower shrinkage than all of AAS compositions regardless of the relative humidity, except for the two 8 M compositions at 75% RH and S05M05 at 55% RH. In the first case, the compositions swell instead of shrinking, while in the latter case the final free strain is the same. In addition, only OPC and 8 M compositions present a distinct increase in the drying shrinkage when reducing the external RH. For the other mortars, it is not possible to establish a common trend; 0.5 M compositions present the lowest shrinkage at 55% RH and a maximum at 75%, while the 2 M ones present the highest shrinkage at 55% RH and the lowest at 75%. The absence of a relationship between drying shrinkage and RH is contrary to what was observed for OPC [28,29] but has already been observed for AAS [6,13]. In addition, increasing the solution-to-binder ratio leads to an increase in the shrinkage regardless of the molarity and relative humidity conditions.

The effect of molarity is not clear either. The vast majority of the literature on the topic agrees that increasing the activator concentration, and therefore the Na_2_O content, increases the shrinkage [11,12,13,21,22,23] due to a higher degree of reaction and a finer pore structure [30]. Even excluding the results for the two 8 M compositions at 75% RH—that will be discussed in more detail in the following paragraphs—the effect of pore refinement and subsequent shrinkage increase is noticeable only at 33 and 55% relative humidity, while at 75% RH it is possible to observe a decrease in shrinkage when increasing the molarity.

Concerning the water-to-binder ratio, its effect on the investigated compositions is not clear. Specifically, the authors expected that a higher water content would have had a bivalent effect; on one hand, it should provide a coarser pore structure and a lower shrinkage, while, on the other hand, a higher quantity of evaporable water [15,31,32]. In this case, the compositions with the same molarity present an increase in shrinkage with the w/b ratio, consistently with the s/b ratio as well. On the contrary, for the compositions with the same solution-to-binder ratio, the shrinkage increases with the w/b ratio only at 75% RH, while the opposite is true for the other two relative humidity conditions, making it impossible to draw a general conclusion.

**Figure 3:**
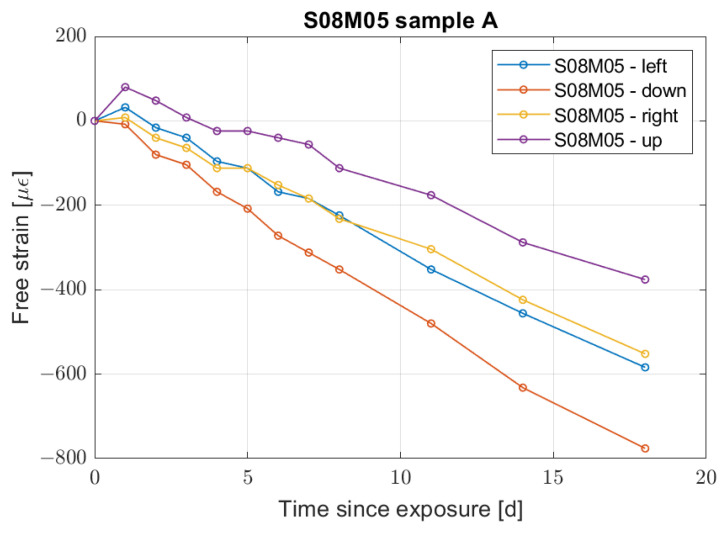
Free strain evolution for sample S4-A according to its side.

As a final remark on the shrinkage behaviour, at early age, composition S08M05 presents a difference in behaviour according to the recorded face of the sample. Figure 3 shows the evolution of sample S08M05-A up to 18 days for the different sides labelled according to their position at the time of casting. It is possible to notice how the lateral sides present a similar shrinkage magnitude, while the upper and the lower sides show a significant difference, meaning the sample is not shrinking in an uniform way and bends slightly towards the bottom side.

**Figure 4:**
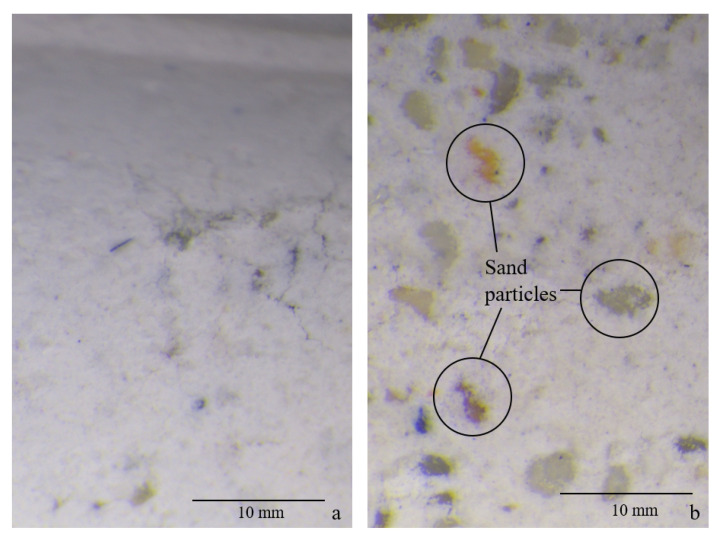
Optical image of S08M05-A’s upper (**a**) and lower (**b**) side.

One possible explanation is that the positioning of the samples inside the RH boxes caused a difference in the drying process, but the absence of said behaviour in the other compositions and the regular turning of the samples on the grid allowed us to exclude this explanation. Another possibility is the occurrence of segregation and bleeding due to the low viscosity of the mortar at the time of casting. This second hypothesis was corroborated by the bleeding observed on the top surface of the samples during the curing time and was verified using a Leica S8 APO optical microscope (Leica Microsystems Belgium BV, Diegem, Belgium) with a DFC camera. Figure 4a,b shows the upper and lower side of sample S08M05-A, respectively. From the figures the presence of more aggregate on the bottom side is clear, with a complete absence of visible sand on the upper side. The bending cause may therefore be the higher content of sand at the bottom of the sample or the higher degree of reaction at the top of the sample due to the higher amount of activating solution.

#### 2.3.2. Autogenous Deformation

**Figure 5:**
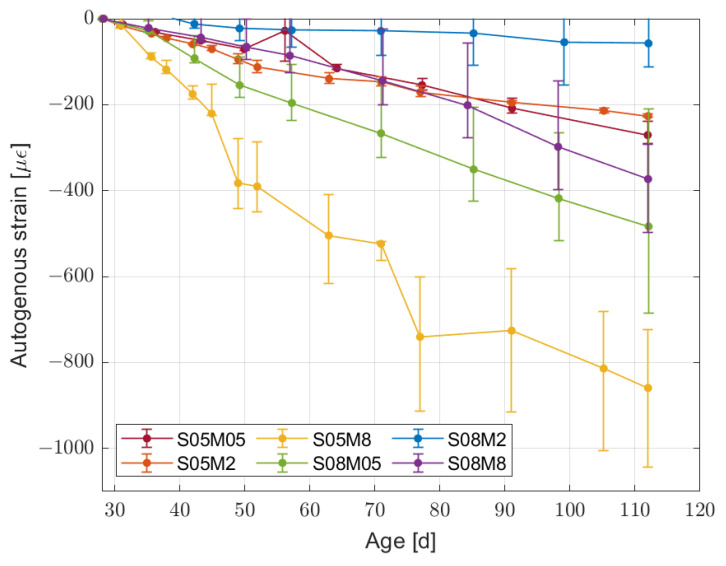
Autogenous deformation for the six compositions initialised at 28 days.

**Figure 6:**
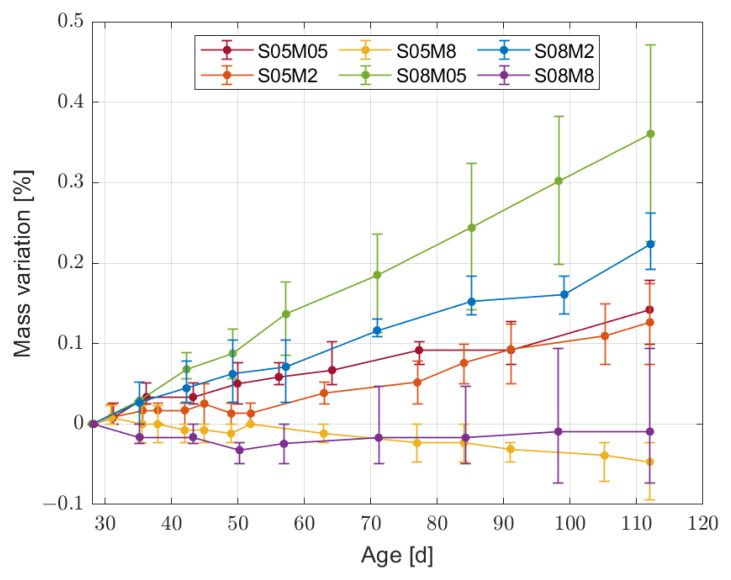
Mass variation of the sealed samples for the six compositions initialised at 28 days.

From the point of view of autogenous deformation, the results up to 112 days are presented in Figure 5. As previously mentioned, the results were initialised at 28 days for comparison purposes. In addition, the mass of the samples was measured regularly in order to ensure a proper sealing of the samples. The mass variation shown in Figure 6 is lower then 0.5% over the 112 days of test, confirming the correct sealing of the samples.

In Figure 5, it is clear that for some compositions the autogenous shrinkage is not negligible. In order to better understand the influence of the autogenous deformation on the total deformation, Table 4 reports the percentage of the total deformation due to the autogenous deformation at 112 days of age.

The first thing to notice is that all compositions at 112 days in drying conditions (corresponding to 84 days after the 28 day curing period) still present an increase in the total deformation when reducing the relative humidity; therefore, the influence of autogenous deformation decreases with the relative humidity as well. For composition S05M05, however, there is a maximum in the influence of autogenous shrinkage on the total shrinkage at 55% RH that already presents a plateau. In addition, the 8 M compositions at 75% RH present a negative value because of swelling.

Developing the discussion in more detail, the highest autogenous deformation takes place for composition S05M8, double of what is observed for composition S08M05. At the same time, the lowest autogenous deformation recorded takes place for composition S08M2. In general, it is not possible to generalise the influence of the molarity or the solution-to-binder ratio on the autogenous shrinkage. It is also important to point out that none of the compositions seem to have reached equilibrium yet; therefore, a longer period of observation may be necessary to observe the emergence of a pattern in the results.

Similarly to what occurs for autogenous deformation alone, its influence on the total deformation does not present a pattern related to the molarity of the solution or its solution-to-binder ratio. Regardless of the short observation period, it is interesting to observe how the autogenous shrinkage magnitude is not an indicator of its influence on the total one; compositions S05M2 and S05M05 present a similar autogenous shrinkage but in the former it represents 46.1% of the total shrinkage at 75% RH against 20.0% in the latter.

#### 2.3.3. Mass Variation

**Figure 7:**
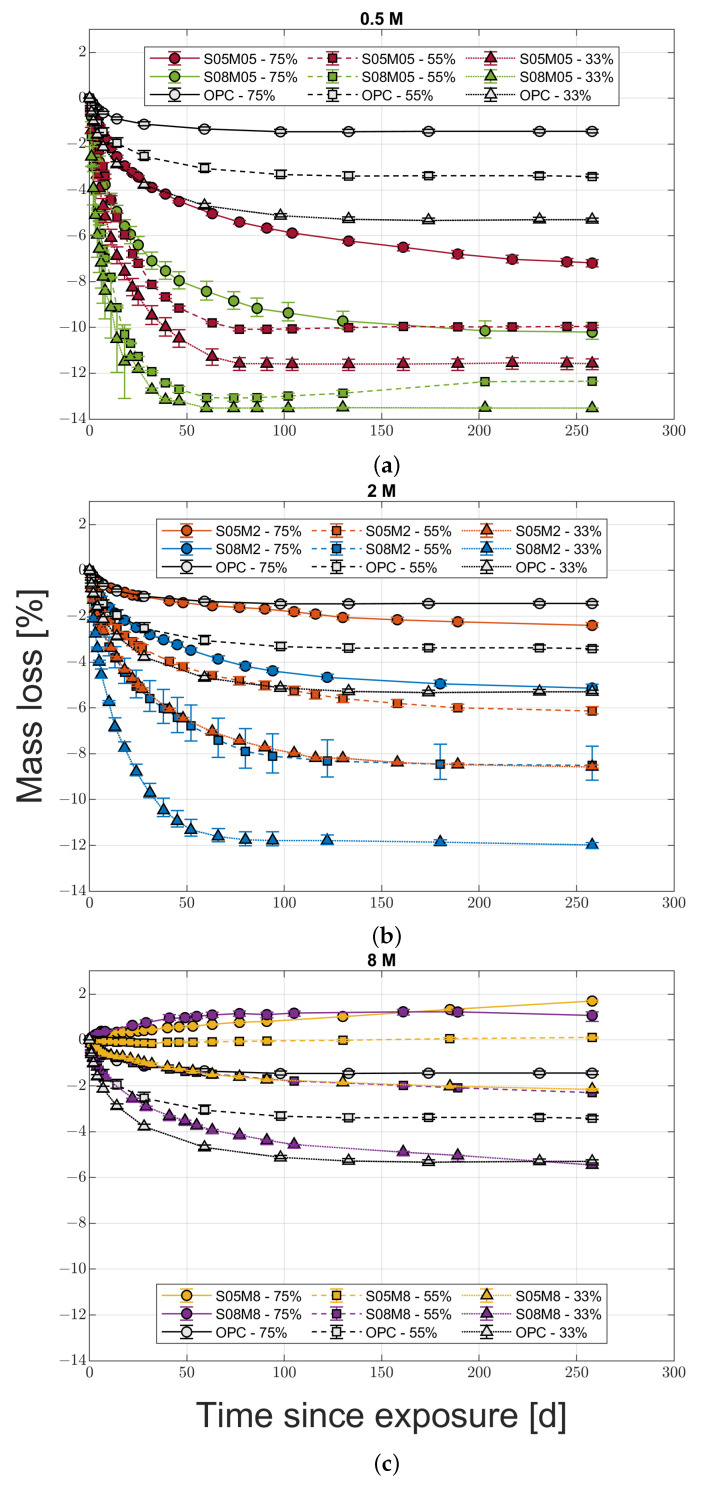
Mass loss evolution for the 0.5 M (**a**), 2 M (**b**), and 8 M (**c**) compositions compared to OPC.

Concerning the mass variation in drying conditions, its evolution over time in the same conditions as the shrinkage ones is presented in Figure 7a–c.

All compositions present an increase in the mass loss when decreasing the RH, in agreement with what has been observed so far for porous materials. Moreover, the mass loss increases when decreasing the molarity, regardless of the solution-to-binder ratio. Finally, both 8 M compositions present a positive mass loss at 75% RH, which represents an increase in the absolute mass of the samples. Similar to what was observed for the shrinkage, increasing the s/b ratio leads to an increase in the mass loss, except for the 8 M compositions at 75% RH, for which S05M08 presents a 60% increase in mass compared to S08M8. The mass increase at high relative humidity values is due to the fact that the initial RH of the compositions is lower than 75%, in line with the available RH values for NaOH solutions at different molarities (58.3% for an 8 M solution at 20 °C) [33]. As a matter of fact, a low initial relative humidity in the samples would also explain the very high autogenous shrinkage observed for composition S05M8.

**Figure 8:**
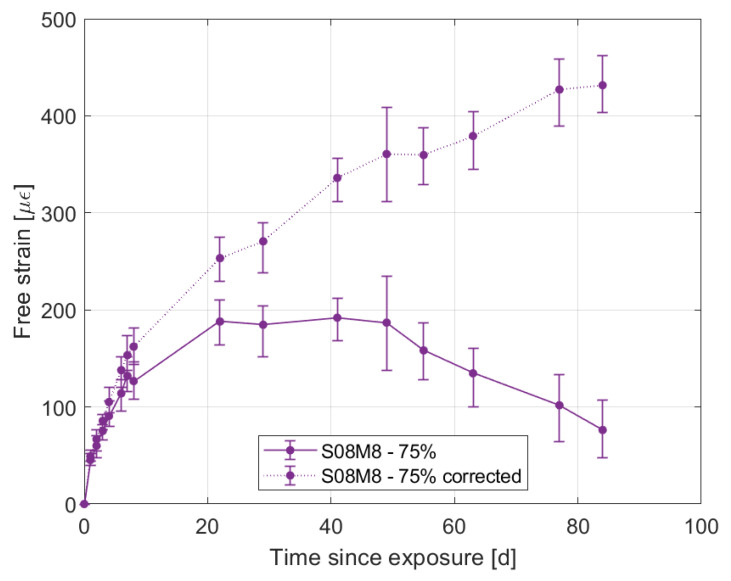
Comparison between the total shrinkage and the drying one for composition S08M8 at 75% relative humidity.

Focusing on the shrinkage and mass loss evolution of 8 M compositions, it is possible to notice how both of them start swelling at 75% RH, in agreement with their mass gain, and then start shrinking even though the samples are still absorbing moisture from the environment or, at least, are in equilibrium with it. There is no occurrence of this phenomenon in the literature. Said behaviour has been confirmed by a second set of samples for S08M8 that confirmed the stability and reliability of the measuring points. A possible reason for the change in behaviour from swelling to shrinkage may be the ongoing autogenous deformation at the moment at which the samples are not absorbing moisture from the environment any more. Specifically, removing the autogenous strain from the total one seems to confirm the still strong influence of the autogenous deformation at 84 days since the drying started, as shown in Figure 8.

**Figure 9:**
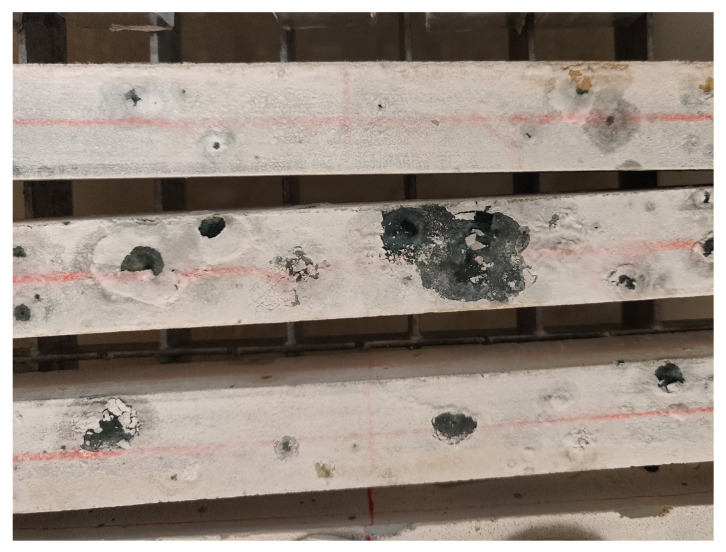
Salt formation on the surface of S08M8 samples at 75% RH.

Another interesting aspect of the 8 M compositions is the salt formation appearing on the sample surface, shown in Figure 9. Specifically, the figure shows samples with a light-coloured surface with darker spots; the light colour is given by the very strong efflorescence that forms a uniform layer on the sample surface. When the salt “crust” brakes, the inner colour of the mortar appears, resulting in the dark spots. The efflorescence is provoked by the high amount of weakly bonded alkalis in the gel structure [34,35], which provides an excess of Na_2_O able to react with the environmental CO_2_ to form Na_2_CO_3_ [36,37,38]. The chemical reaction on the surface is responsible for a concentration gradient inside the sample, which forces more Na_2_O towards the surface and feeds the process. Conversely, the effects of said phenomenon on the material volume stability are not clear, as a deeper investigation is necessary to understand this specific phenomenon.

Concerning the other compositions, the effect of the relative humidity on the drying shrinkage magnitude is unexpected if compared with standard OPC mortars, and not so much so if compared to other studies on alkali-activated slag. Specifically, Ye and Radlińska [6] attribute the higher shrinkage in higher RH conditions to a microstructural rearrangement of C-A-S-H particles in the material. In addition, nitrogen and semi-equilibrium water sorption isotherm data confirm that said refinement would be responsible for the collapse of gel pores and refinement of the pore structure in general. In addition, the authors noticed how plotting the drying shrinkage against the mass loss provides an almost linear relationship for OPC regardless of the relative humidity, while this was not true for alkali-activated slag.

#### 2.3.4. Mass Loss Versus Drying Shrinkage

**Figure 10:**
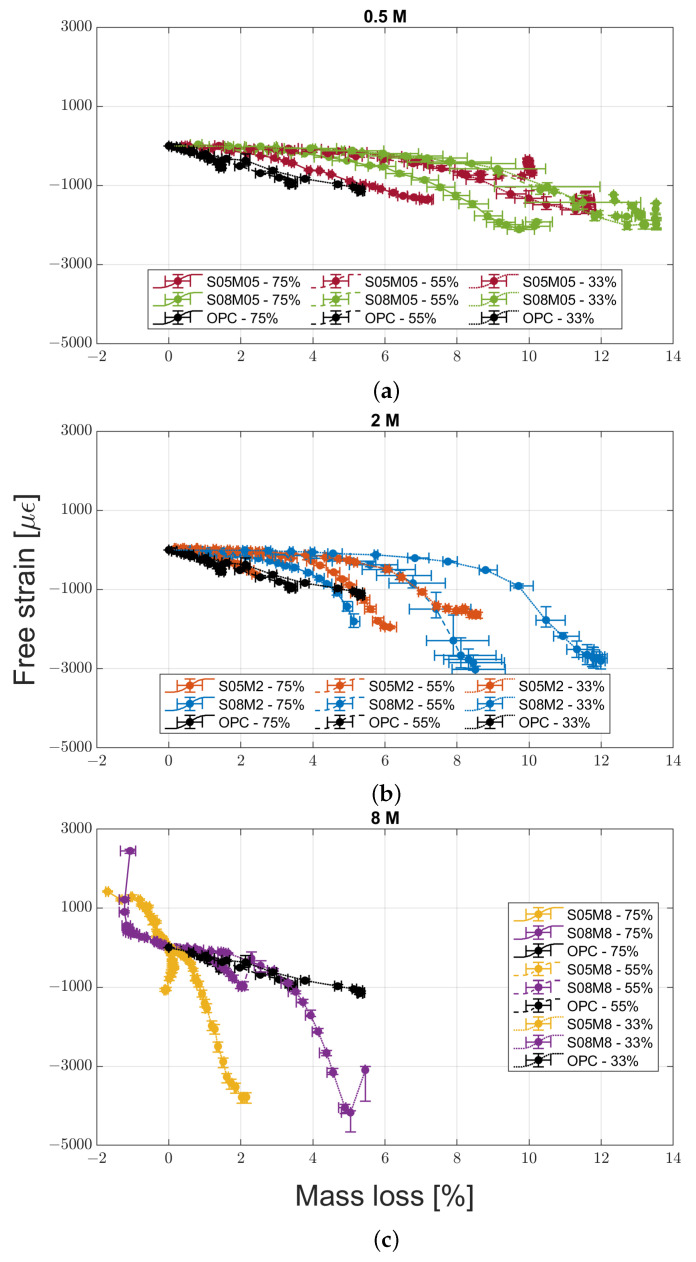
Drying shrinkage over mass loss for the 0.5 M (**a**), 2 M (**b**), and 8 M (**c**) compositions compared to OPC.

In this study, a comparison between mass loss and shrinkage [6] is provided in Figure 10a–c.

In this case, it is possible to define the strain as drying shrinkage as the autogenous component has been removed from the total strain. In addition, in order to account for the age difference, the autogenous shrinkage data have been extrapolated to match the total shrinkage age. Specifically, the autogenous shrinkage follows a logarithmic trend and fitting was performed using the least square method, which allowed for a good agreement with the experimental data. Specifically, the R^2^ of the fitted line is always ≥0.93. From the point of view of the results, most compositions present swelling when the samples reach equilibrium or present very little mass variation. As a matter of fact, said swelling is just the product of removing the still evolving autogenous strain from the already constant total strain, while the material presents only negligible deformation. A possible reason is that the drying process removed all available water, impeding further self-dessication. The experimental data presenting artificial swelling were removed from the dataset and used for further computation and discussion.

**Figure 11:**
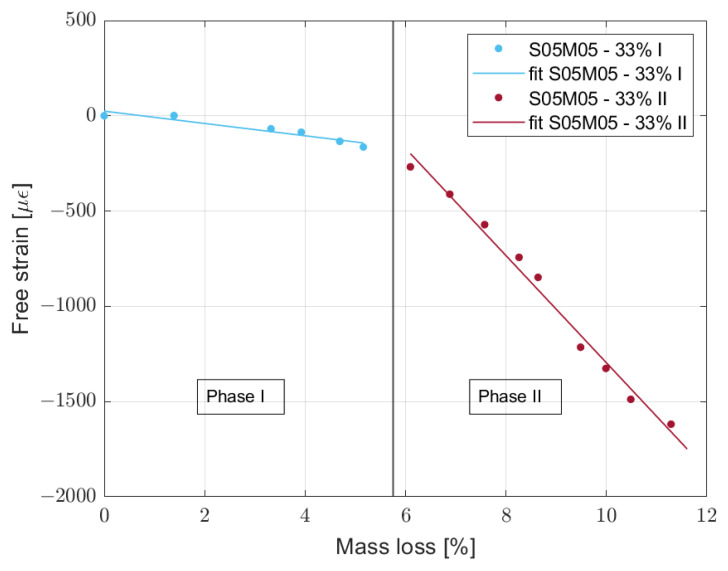
Drying shrinkage over mass loss for S05M05 dried at 33% relative humidity with the segmented fitting and phase distinction.

Moreover, it is interesting to notice that most of the experimental data can be fitted using a segmented regression model. Specifically, it is possible to use two linear equations for the same curve that fit the part before and after its knee. For the sake of simplicity, phase I is the part before the knee and phase II the part after it. A clear example is composition S05M2 dried at 33% relative humidity, shown in Figure 11. The only cases that do not follow said behaviour are S05M8 dried at 55 and 75% RH and S08M8 dried at 75% RH. During phase I, the material loses water while the volume does not change much, while during phase II, the shrinkage increases much faster than during phase I. A high value of the transport properties of the material would explain the change in the drying behaviour. If the water has high mobility inside the material, the external layers of the sample are able to dry very fast while the core does not have such a possibility. As a consequence, the inner part of the sample acts as a restraining factor, hindering the registered volume change, while microcracks appear in the external layers that then dry even faster [39,40,41]. In order to verify said hypothesis, further studies are needed, as the water transport properties of alkali-activated slag are still unknown.

The segmented regression model is based on the least square method and a summary for the different compositions is presented in Table 5. The delay represents the change in slope as a function of time after the exposure to the external RH obtained as the intersection of the two fitted lines. The absence of values for the 8 M compositions is caused by the impossibility to fit the data due to the change in behaviour of the compositions between shrinkage and swelling.

Going into the details of the values reported in Table 5, all compositions except S05M2 present the longest delay at 75% RH, which would be the expression of a change in the speed of drying with the relative humidity. In addition, reducing the relative humidity also reduces the delay, except for S05M05, which presents a slightly longer delay at 33% RH compared to 55% RH. As a consequence, we can assume that the drying speed increases when decreasing the relative humidity, without presenting a maximum or minimum value between 33 and 75% RH, for both 2 and 0.5 M compositions. In order to verify the effect of the relative humidity on the hydraulic conductivity of alkali-activated slag, further investigation is required as the absence of any study on the topic makes it impossible to verify or disprove said hypothesis. Concerning the slope of phase I, it is always lowest at 75% RH, meaning that the limitation provided by the wetter core is less relevant than in the other conditions. The results are then in line with the hypothesis of a slower drying process. However, the slopes of phase II do not present any relationship with the change in relative humidity; however, for 0.5 and 2 M compositions, the higher the slope the higher the final shrinkage value, except for composition S08M2 at 75% RH. Said correlation is not surprising as the slope represents how fast the shrinkage evolves compared to the mass loss. At the same time, a longer monitoring would probably allow composition S08M2 to reach equilibrium and present a higher shrinkage in line with the slope observed so far, even though the mass loss is already very low.

To conclude the comparison between mass loss and drying shrinkage, if for OPC there is a clear difference between the RH values, for most AAS compositions this is not the case. For S05M05 in Figure 2, it is particularly evident, as the final shrinkage at 75% RH is the same as the one at 33% and double of the one at 55%. In order to understand why the composition presents such behaviour, two main hypotheses were taken into account. The first one is that the lower drying rate at 75% RH is responsible for a longer permanence in a state of tension caused by the shrinkage mechanisms, such as capillary pressure, disjoining pressure, and surface free energy [13,42]. If the samples are exposed to internal forces for a longer period of time, it is possible for them to present a higher creep deformation, as it is known that in OPC there is a linear relationship between drying shrinkage and creep [43,44]. The second hypothesis is that the formation of microcracks is more pronounced for the samples exposed to lower relative humidity conditions; the steeper internal RH gradient causes the appearance of stronger stress in the sample and subsequently more microcracking [45]. The presence of microcracks allows for a faster drying process and partial relief of the internal stress, therefore reducing the shrinkage [46,47]. Both hypotheses have been checked with specific tests that will be presented in the following sections.

## 3. Effect of Water Content on Creep

Creep is defined as the deformation of the material under sustained loading and it represents one of the main factors affecting the durability of cementitious materials. Even though the mechanisms of creep are not fully understood yet, it is possible to attribute it to three main phenomena, namely water transport, viscous shear, and microcracking [48,49]. Without entering the details of said mechanisms, as the water movement affects the creep deformation of cement, it is reasonable to assume that the water content or the water state of the material has a relevant effect on the creep deformation. As a matter of fact, studies conducted on OPC in drying conditions showed that the creep increases when decreasing the external relative humidity [48]. In addition, shrinkage-induced microcracking also seems to play a role in the creep behaviour of cement [50], even though the microcracking itself is cancelled by the external load [51]. Finally, concretes showing high shrinkage also show high creep [48]. For alkali-activated slag, the only available study shows that the creep coefficient increases by around 70% from sealed to drying conditions [52].

The goal of testing the creep deformation in partially dried samples is to understand how the difference in water content in the samples dried under different conditions affects the creep of alkali-activated slag. As a matter of fact, the change in creep magnitude may also be responsible for the higher shrinkage in high relative humidity conditions observed for certain compositions.

### 3.1. Mortar Composition

The test was carried out for composition S05M05 from Table 2, for which creep may increase the recorded shrinkage magnitude in high relative humidity conditions.

### 3.2. Experimental Procedure

#### Creep Test

**Figure 12:**
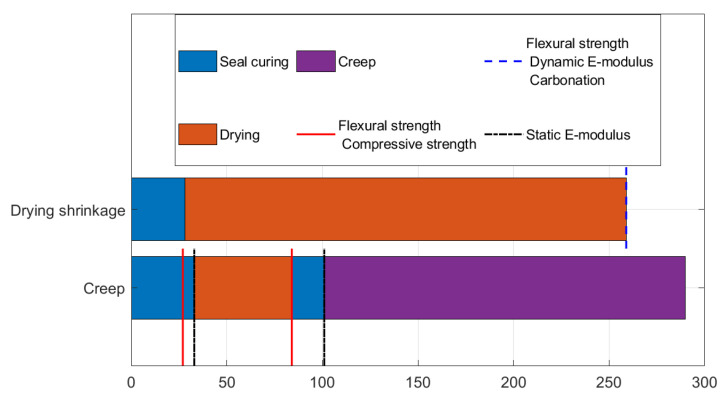
Curing and test scheme for the different experimental campaigns.

The samples used for the test were prisms with a square cross section of 40 mm and length of 160 mm. As the test aims to study the effect of water content on creep, the curing conditions should replicate the drying history of the previous tests. In addition, measuring the creep in drying conditions would not take into account the different RH values nor would it allow for a constant water content, for which it is easier to understand the results. For the aforementioned reasons, the samples were seal-cured for 33 days, exposed to drying at 33, 55, and 75% RH for 51 days, and then sealed again with a double layer of aluminium. In addition, the samples were then seal-cured for another 17 days to allow a reduction in the internal relative humidity gradient before starting the test and applying an external load. The timeline of the experimental campaign is outlined in Figure 12. The drying period of 51 days allowed for a clear difference in the water content of the samples exposed to different RH levels, while the subsequent 17 days in sealed conditions were chosen arbitrarily, as no direct measurement of the internal relative humidity in different positions was performed. As a comparison, the same composition was tested at the same age in sealed conditions.

**Figure 13:**
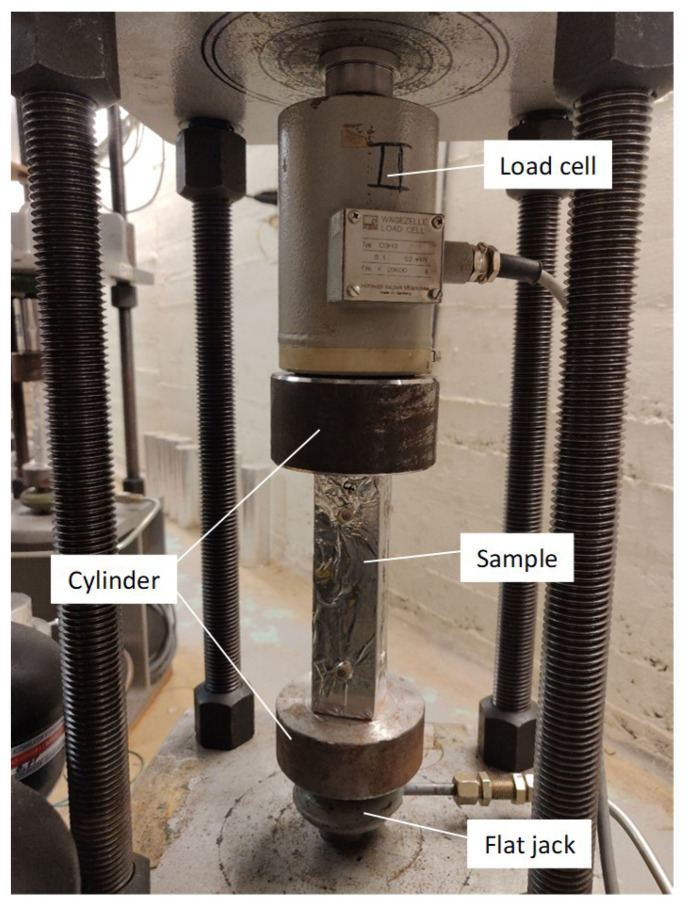
Compressive creep rig.

The external load applied corresponds to 20% of the compressive strength measured before the test, namely 2.5 MPa, and is kept constant over time. The creep test rig is shown in Figure 13. The main components of the equipment are the load cell, the flat jack, and the steel cylinder. Specifically, the load cell is a sensor that measures the force applied to the sample, while the flat jack is the piece applying the load to the sample; the flat jack is filled with oil and is able to deform according to its inner pressure and therefore apply a force to the sample. Finally, the steel cylinder can be considered as a transition piece to ensure a correct application of the force to the specimen. To load the sample, a hydraulic group increases or decreases the pressure of the oil filling the flat jack, while the load cell provides feedback on the actual force experienced by the sample. The centring of the specimen proved particularly complicated due to the small size of the prisms, but all samples showed pure compression when loaded, guaranteeing the absence of tension.

The deformation was measured using a DEMEC with a gauge length of 100 mm and a resolution of 15.6 με on two different samples per relative humidity condition and on two sealed samples. In addition, the test also includes the length change for two dummy samples cured in the exact same conditions but without any load applied, in order to remove autogenous deformation from the measured displacement. In addition, in this case, two samples were tested for each curing condition.

Finally, the creep compliance was calculated according to the following equation:

(1) $$J=\frac{\left(\varepsilon_{c}-\varepsilon_{s}\right)}{F/A}$$

where *J* is the creep compliance ($\frac{\mu \varepsilon }{\mathrm{MPa}}$), $\varepsilon_{c}$ is the deformation of the loaded samples (με), $\varepsilon_{s}$ is the deformation of the dummy samples (με), *F* is the force applied (*N*), and *A* is the surface of the sample where the load is applied (m^2^).

### 3.3. Results and Discussion

**Figure 14:**
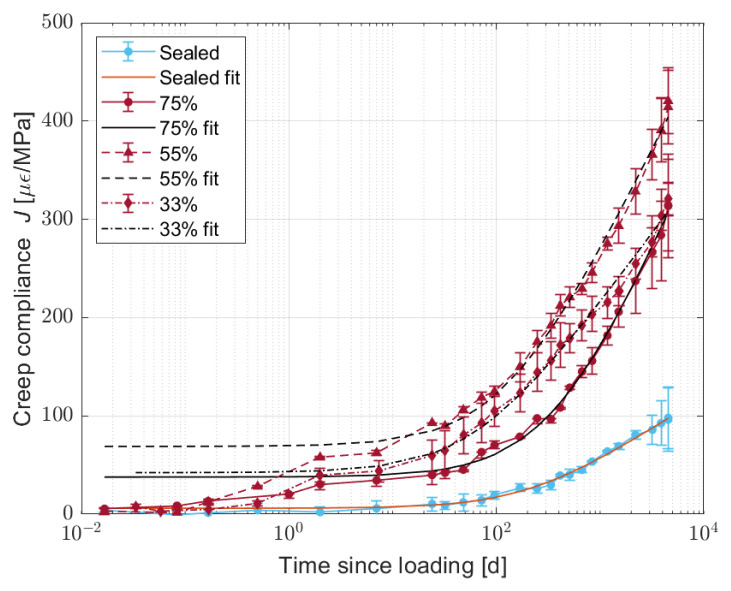
Creep evolution for sealed and partially dried S05M05 samples at 33, 55, and 75% relative humidity.

The average mass losses of the creep samples after 51 days of drying are equal to 7.90, 6.40, and 3.03% at 33, 55, and 75% RH, respectively. Even though these values correspond to 18–22 days of drying, for the thinner drying shrinkage samples, the authors considered that the difference in water content was enough to evaluate its effect on the creep deformation. The averages for each face of the sample and for the different samples are shown in Figure 14.

From Figure 14, it is possible to observe that the partially dried samples present a fast increase in the creep compliance, followed by a slower evolution that follows a logarithmic trend, already observed for OPC [53]. Conversely, the sealed samples present only the logarithmic trend.

The fast initial increase in the creep compliance could be caused by the movement of water inside the specimen, meaning that 17 days of curing in sealed conditions was not enough to completely stabilise the water content in the specimen. Comparing the sealed and dried samples, the creep compliance magnitude for partially dried samples is at least 1.5 times higher than that for sealed samples. In addition, among the dried samples, the highest creep deformation occurs in samples dried at 55% RH, the same one that showed the lowest drying shrinkage, allowing us the exclude creep as the reason for why the drying shrinkage is highest at 75% RH. On the other hand, the higher creep compliance at 55% RH could represent a higher relaxation of the stress and a subsequent reduction in the drying shrinkage.

The fitting of the experimental results was carried out using the least square method for the following equation:

(2) $$J=A+\frac{1}{C}\log\left(1+\frac{t-t^{\prime }}{\tau }\right)$$

where *A* is a constant that considers the initial increase in creep compliance for the partially dried samples ($\frac{\mu \varepsilon }{\mathrm{MPa}\;}$), *C* is an amplitude parameter ($\frac{\mathrm{MPa}}{\mu \varepsilon }$), *t* and $t^{\prime }$ are the age at loading and the time since loading (d), respectively, and τ is a kinetic parameter (d).

**Figure 15:**
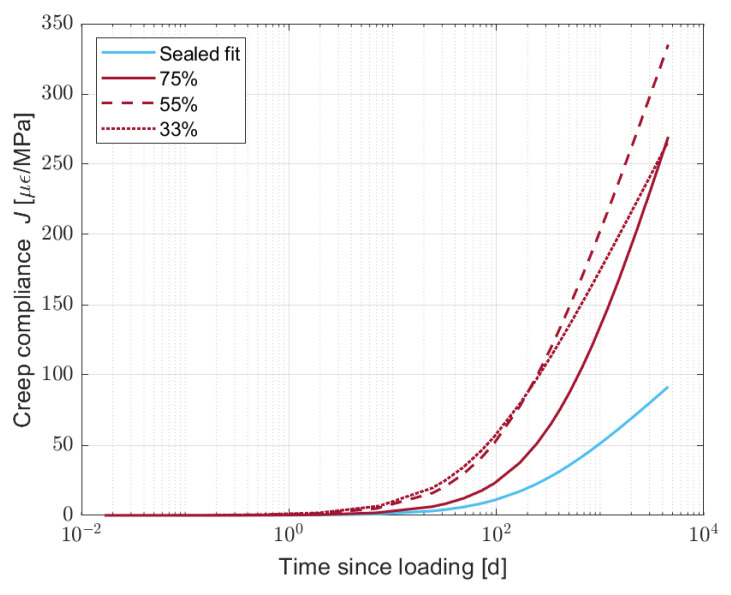
Creep fitted curves after removing the initial water movement for sealed and partially dried S05M05 samples at 33, 55, and 75% relative humidity.

As the initial and fast increase in the creep compliance could be induced by the water movement, it is more interesting to remove it from the final results in order to properly compare the effect of the water content on the creep behaviour. Figure 15 shows the fitted curves for the creep after removing the *A* factor in Equation (2). Specifically, the water movement deformation takes place during the first week after applying the load. Table 6 shows the values of *A*, *C*, and τ for the four different curves.

In Figure 15, it is possible to observe that the samples cured at 33% RH present a gentler slope when compared to the other two partially dried cases, as illustrated by the higher *C* factor. In addition, the samples dried at 55 and 75% RH present a very similar slope but with a different delay for the samples cured at 75% RH. Said delay is well represented by τ, which decreases with the relative humidity and at 75% is more then three times higher than that at 55%.

**Figure 16:**
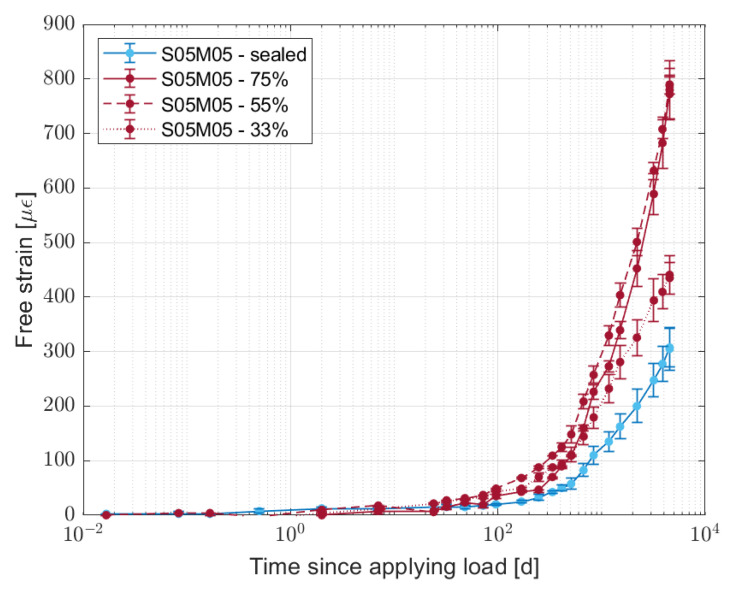
Shrinkage evolution for S05M05 dummy samples, sealed and partially dried at 33, 55, and 75% relative humidity.

The average shrinkage value for the dummy samples is shown in Figure 16. In this case, the average is computed on the four faces of the sample and the two different samples tested.

For the partially dried samples, the results of the shrinkage deformation of the dummy samples show the same behaviour observed for the creep compliance; the highest deformation takes place for the samples dried at 55% RH, 1.5 times higher than what was observed for the samples dried at 33% RH. As the age of the samples is the same regardless of the curing history, the only cause of the difference in shrinkage values is the water transport inside the samples. As the relative humidity is not uniform in the samples, it is difficult to compare the results for the creep test with what was observed for the dummy samples, as in the first case the internal RH is made uniform mechanically. In order to overcome such an issue, it would be interesting to apply a load on the dummy samples as well at the beginning of the test in order to remove the effect of a non-homogenous internal relative humidity.

## 4. Flexural Strength, E-Modulus, and Carbonation

It is known that one of the main effects of drying shrinkage is the formation of microcracks in the material [11,15,54,55]. In addition, the presence of microcracks in cementitious materials has been proven to affect their transport and mechanical properties. Specifically, the presence of microcracks increases the permeability and diffusivity of the material and accelerates the drying process and the penetration of external chemical agents such as CO_2_ [45,56,57]. From a mechanical point of view, the microcracking process reduces the compressive strength, tensile strength, E-modulus, and Ultrasonic Pulse Velocity (UPV) [58,59,60]. For these reasons, it is possible to estimate the amount of microcracks in a specimen under drying conditions by comparing its mechanical properties with a sealed reference. In the present study, the comparison was carried out for composition S05M05 dried at different relative humidity values considering the flexural strength, the E-modulus, and the carbonation depth.

From the point of view carbonation, already existing studies on NaOH-activated slag show the formation of calcite, aragonite, vaterite, and natron in the microstructure [16,61]. Moreover, a decrease in the hydrotalcite phase is observed, as it acts as a sorbent of CO_2_ [61]. In addition, both MIP and SEM images exhibited a higher matrix density with a refinement of the pore structure [16]. Finally, FTIR tests confirmed the formation of calcite but also indicated a higher degree of polymerisation [61].

### 4.1. Mortar Composition

The tested composition is S05M05 from Table 2, both in drying and sealed conditions. The samples have the same geometry and curing conditions as the ones used for creep. The compressive and flexural strengths were tested at 27 and 84 days of age, while the E-modulus was tested at 33 and 101 days of age, before starting the drying curing of the samples and at the start of the creep test, respectively.

In parallel, the flexural strength, dynamic E-modulus, and carbonation depth were tested for the same composition under drying and sealed conditions but on the same samples used to measure the drying shrinkage, while the sealed samples were cast specifically for the test. The dried samples were tested at an age of 315 days, while the sealed ones were tested at an age of 107 days.

### 4.2. Experimental Procedure

#### 4.2.1. Flexural Strength

The flexural and compressive strength of the 40 × 40 × 160 mm samples was tested using a Walter+bai ag hydraulic test machine (walter + bai Testing Machines, Löhningen, Switzerland) with a capacity of 10 kN for the flexural strength and 200 kN for the compressive strength.

The flexural strength of the 25 × 25 × 285 mm samples was obtained from a three-point bending test using a Walter+bai hydraulic press with a capacity of 40 kN. The test velocity was set to 10 N/s and the distance between the supports was 100 mm. Finally, the strength was calculated using the following equation:

(3) $$\sigma_{f}=\frac{3\times F\times L}{2\times w\times h^{2}}$$

where *F* is the measured force (kN), *L* is the support span (m), *w* is the width of the sample (m), and *h* is the thickness of the sample (m).

#### 4.2.2. Static E-Modulus

The static E-modulus was determined twice for each sample while applying two different forces of 0.2 and 4.1 kN in order to verify the repeatability of the test, according to the European Standard [62]. The load was applied using a hydraulic press. Specifically, 4.1 kN corresponds to 20% of the compressive strength, in order to measure only the elastic behaviour of the material. The instrument used for measuring the static E-modulus was, once again, a DEMEC with a gauge length of 100 mm and a resolution of 15.6 με. In this case, the equation used for the computation of the secant E-modulus *E* is:

(4) $$E=\frac{F_{1}-F_{2}}{A\times (\varepsilon_{1}-\varepsilon_{2})}$$

where $F_{1}$ and $F_{2}$ are the forces applied during the test (kN), corresponding to 0.2 and 4.1 kN, respectively, *A* is the area of the sample (m^2^), and $\varepsilon_{1}-\varepsilon_{2}$ is the deformation measured with the DEMEC (με).

#### 4.2.3. Dynamic E-Modulus

The dynamic E-modulus was determined using a Procecq Pundit Lab+ UPV instrument (Screening Eagle Technologies AG, Schwerzenbach-Zurich, Switzerland) that measures the time needed for the ultrasonic pressure waves to cross the specimen. Specifically, it was measured at two different points of the sample and the elastic modulus was then computed according to the following equation:

(5) $$E_{\mathrm{HF}}=V_{p}^{2}\times \rho \times \frac{\left(1+\nu_{HF}\right)\times \left(1-2\times \nu_{HF}\right)}{\left(1-\nu_{HF}\right)}$$

where $V_{p}$ is the measured *p*-wave velocity (m/s), ρ is the density of the material (kg/m^3^), and $\nu_{\mathrm{HF}}$ is Poisson’s ratio. Specifically, the density is equal to 2.15 kg/m^3^ and Poisson’s ratio is assumed equal to 0.15 [63].

#### 4.2.4. Carbonation Depth

The carbonation depth of the dried specimens was measured using phenolphthalein and a calibre, according to standard BS EN 14630-2006 [64]. Due to the small size of the samples, only three measurement per side were taken.

### 4.3. Results and Discussion

#### 4.3.1. Compressive Strength

**Figure 17:**
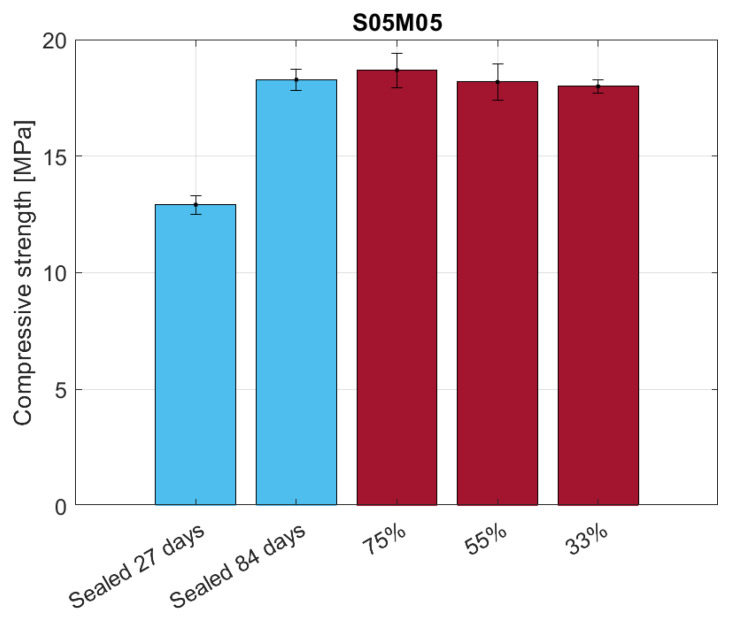
Compressive strength of S05M05 under sealed and drying conditions.

Figure 17 shows the results for the compressive strength. In sealed conditions, it is possible to observe an increase in the strength due to the ageing of the material, while no significant difference appears for the dried samples.

#### 4.3.2. Flexural Strength

**Figure 18:**
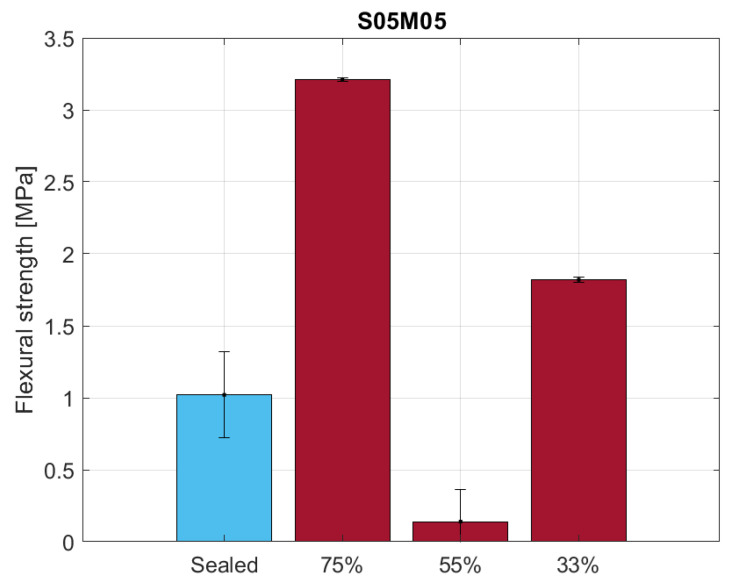
Flexural strength of S05M05 in sealed and drying conditions for 25 × 25 × 285 mm samples.

**Figure 19:**
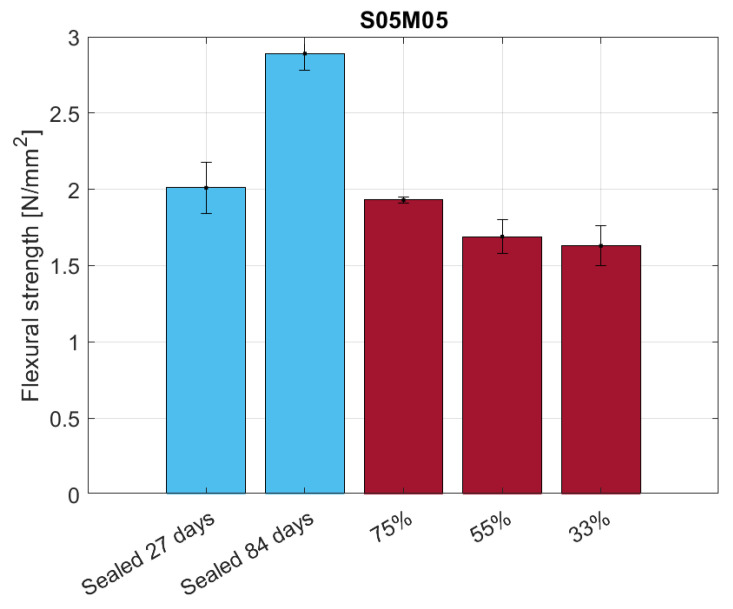
Flexural strength of S05M05 in sealed and drying conditions for 40 × 40 × 160 mm samples.

The flexural strength results for the 25 × 25 × 285 mm samples are shown in Figure 18. Even though preliminary tests did not show any significant ageing effect on the material for such thin samples, the comparison between dried and sealed conditions suggests there is such an effect in the long term. Nevertheless, what is significant from the comparison is the effect of the relative humidity on the flexural strength of the samples as the minimum is reached at 55% RH. As the loss in mechanical properties can be attributed mostly to the presence of microcracking, the authors expected a decreasing flexural strength with the relative humidity conditions [65]. Nonetheless, the high presence of microcracks at 55% RH agrees with the low drying shrinkage observed [66]; therefore, the presence of microcracks can be considered as the main cause of the unexpected drying shrinkage behaviour of alkali-activated slag when compared to OPC. The same test performed under partially dried conditions on thicker samples shows a different behaviour, as presented in Figure 19. In this case, the results of the sealed samples show the presence of an ageing effect and also a behaviour closer to what we would expect for dried samples, as the minimum flexural strength occurs at 33% RH. The discrepancy between the two tests can be attributed to the level of drying of the samples; as the samples used for the creep test are thicker, they take more time to reach the same degree of saturation observed in the thinner samples, therefore requiring a longer drying period to observe the same degree of microcracking in the material.

#### 4.3.3. Dynamic and Static E-Modulus

**Figure 20:**
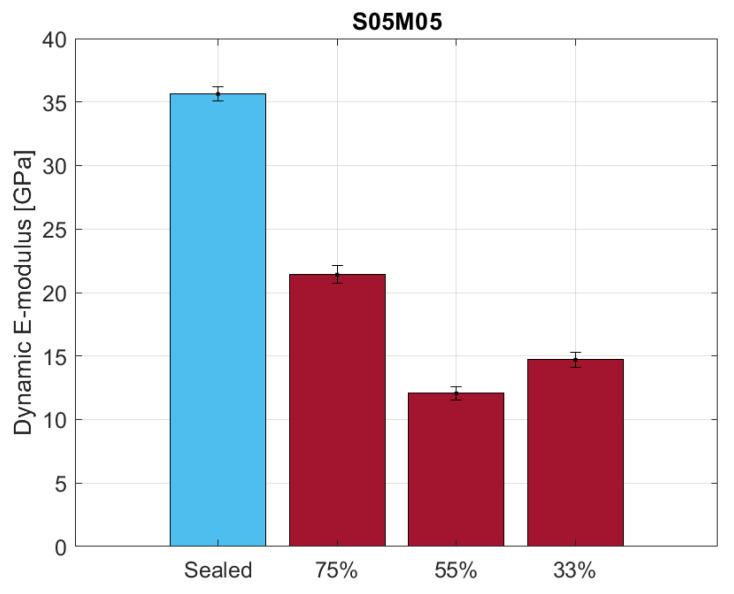
Dynamic E-modulus for S05M05 under sealed and drying conditions using the UPV method.

Figure 20 shows the dynamic E-modulus results of the UPV test for S05M05 under sealed and drying conditions for thin samples of 25 × 25 × 285 mm. The results confirm what was observed for the flexural strength under the same conditions, corroborating the hypothesis of a higher presence of microcracks in the samples dried at 55% RH. In addition, it is also possible to compute the damage factor *d* of the sample using the following equation [67]:

(6) $$d=\left(1-\frac{E_{d}}{E_{s}}\right)$$

where $E_{d}$ and $E_{s}$ are the E-modulus values under drying and sealed conditions, respectively. For the different RH values, the damage factors are 0.59, 0.66, and 0.40 at 33, 55, and 75% RH, respectively. Due to the age effect on the mechanical properties and the age difference between dried and sealed samples, the damage factor values have a purely comparative purpose and are not reliable.

**Figure 21:**
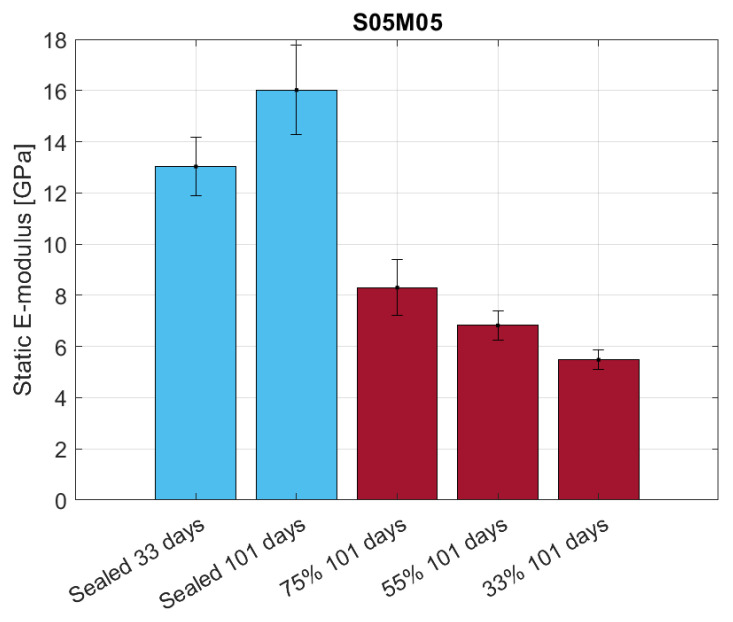
Static E-modulus for S05M05 under sealed and drying conditions.

The static E-modulus measured using the DEMEC on 40 × 40 × 160 mm samples presents the same characteristic as the flexural strength and the results are shown in Figure 21. In this case, the damage factor values are 0.66, 0.57, and 0.48 at 33, 55, and 75% RH, respectively, reflecting E-modulus evolution with the relative humidity.

#### 4.3.4. Carbonation Depth

**Figure 22:**
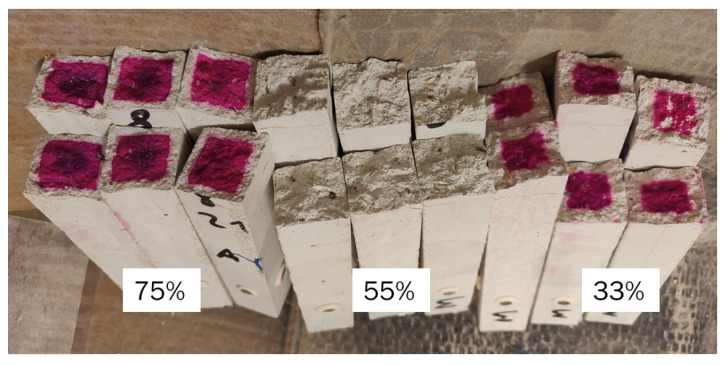
Carbonation depth for S05M05 at 75 (**left**), 55 (**center**), and 33% (**right**) relative humidity.

Tests of the carbonation depth using phenolphthalein exhibited very interesting results. Figure 22 presents a picture of the different samples after the application of the chemical reactant. It is possible to observe that the samples dried at 55% RH show no magenta in the inner part of the sample, as occurs for the two other cases, meaning that the samples are fully carbonated to the core. Specifically, the average carbonation depth is 4.84 and 2.95 mm at 33 and 75% RH, respectively. From the literature on OPC, we know that the maximum carbonation rate takes place around 55% RH [68,69] due to the optimal conditions of diffusivity and the presence of a dissolution interface, while for NaOH-activated slag, the maximum carbonation rate occurs around 65% RH [70]. The results are then closer to what was observed for OPC than for AAS, possibly due to the higher presence of microcracks observed during the flexural strength and dynamic E-modulus tests. In addition, in this case, we observed a minimum in the carbonation depth at 75% instead of 33% RH as we would expect. The reason for this discrepancy is the presence of microcracks, as they act as a preferred route for the CO_2_ to penetrate into the inner part of the material; CO_2_ diffusion is 10^4^ times higher in air than in water [71].

One important thing to bear in mind about carbonation is that in Ordinary Portland Cement, CO_2_ reacts with Ca(OH)_2_ or portlandite first and then with the C-S-H gel. On the contrary, alkali-activated slag does not contain a portlandite phase, meaning that the first affected phase is the C-A-S-H or N-A-S-H gel [71]. FTIR and XRD studies on the carbonated microstructure of NaOH-activated slag confirmed a reduction in the amount of C-A-S-H and N-A-S-H gels due to the interaction with atmospheric carbon oxide [61].

The effect of carbonation on the microcracking and shrinkage of alkali-activated materials is still not clear; a study by Puertas et al. [16] shows a clear refinement of the pore structure of NaOH–slag systems, which should increase the shrinkage magnitude, while Ye and Radlińska [17] observed how the formation and growth of crystalline products of carbonation inside the pores cause expansion and microcracking in the material, making the effects of pore refinement negligible. Contrary to both previous studies, Humad et al. [72] observed a coarsening of the pore structure in the carbonated areas of the samples and an increase in the drying shrinkage. Nevertheless, it is clear that the carbonation process is highly enhanced by microcrack formation caused by drying shrinkage [16], even though the absence of a non-carbonated reference makes it impossible to clearly establish the effects of carbonation on the volume stability of the material and a deeper investigation is needed to better understand them.

## 5. Conclusions

The study of drying shrinkage of alkali-activated slag with all the subsequent tests on creep, mechanical properties, and carbonation has proven challenging and presented results very different from those observed for OPC. In addition, quantitative testing also provided new experimental evidence regarding the alkali activation of the slag itself, as it showed how changing the composition has different effects according to the external conditions. The main conclusions can be summarised as follows:

- Increasing the molarity increases the drying shrinkage of the material only in medium to low relative humidity conditions, while the opposite is true for high RH conditions.
- For the different compositions, the mass loss is not a good indicator of the shrinkage magnitude, contrary to what has been observed for OPC so far.
- Increasing the solution-to-binder ratio increases the drying shrinkage and the mass loss regardless of the molarity and the relative humidity, indicating that the water content affects the drying shrinkage more than the coarsening of the pore structure, at least for the compositions studied.
- High molarity compositions at high relative humidity present an initial swelling due to water vapour absorption, with a subsequent shrinkage that does not agree with the mass stabilisation or increase observed. The cause for this phenomenon is likely to be the autogenous deformation that is still ongoing. In addition, they also present strong efflorescence that forms a salt layer on the surface of the samples.
- The influence of autogenous shrinkage on the total deformation is not negligible even after 84 days. The autogenous shrinkage itself does not seem to follow any pattern linked to the molarity or the solution-to-binder ratio.
- Compositions with a low molarity and a high solution-to-binder ratio present bending caused by the segregation taking place at the time of casting.
- Reducing the amount of water in the material increases the creep compliance compared to the sealed condition, even though the results do not present a clear relationship with the mass loss or the relative humidity or with the change in the mechanical properties. Further investigation is needed in order to understand how the water content of the material affects its creep compliance.
- The minimum shrinkage deformation for the composition with low molarity and a low solution-to-binder ratio occurs at 55% RH due to the higher presence of microcracks, which was observed when testing the flexural strength and the dynamic E-modulus using the UPV method.
- The carbonation depth presents a maximum at 55% relative humidity, as is the case for OPC, even though the cause seems to be the high presence of microcracks that facilitate the penetration of CO_2_ in the material, rather than the optimal conditions of diffusivity and the presence of a high dissolution interface. In order to understand the effect of carbonation on the volume stability, further investigation is required.

## Figures and Tables

**Table 1:** Oxide composition of the slag [25].

Oxide	SiO_2_	Al_2_O_3_	Fe_2_O_3_	CaO	MgO	K_2_O
**Content (%)**	36.2	12.4	0.6	39.8	7.3	0.5

**Table 2:** Mortar mix proportions.

Composition	Solution-to-Binder Ratio	NaOH Concentration (M)	Sand-to-Paste Ratio	Water-to-Binder Ratio
S05M05	0.5	0.5	1	0.49
S05M2		2		0.44
S05M8		8		0.29
S08M05	0.8	0.5		0.77
S08M2		2		0.69
S08M8		8		0.43

**Table 3:** Saturated salt solutions and relative humidity [27].

**Saturated Salt Solution**	**Temperature (** ° **C)**				
	10	15	20	25	30
	**RH over the Salt Solution (%)**				
Magnesium chloride—MgCl_2_	33	33	33	33	32
Magnesium nitrate—Mg(NO_3_)_2_	57	56	54	53	51
Sodium chloride—NaCl	76	76	75	75	75

**Table 4:** Percentage of total deformation due to autogenous deformation at 112 days of age.

Composition	33%	55%	75%
S05M05	13.1%	28.2%	20.0%
S05M2	17.1%	22.1%	46.1%
S05M8	21.9%	54.8%	−336.5%
S08M05	19.5%	22.1%	20.0%
S08M2	2.0%	2.3%	7.0%
S08M8	13.8%	35.4%	−595.1%

**Table 5:** Delay and slope for the segmented regression model used to fit the comparison between mass loss and free strain for the six different AAS compositions exposed to 33, 55, and 75% relative humidity.

Composition	RH	Delay (d)	Slope Phase I	Slope Phase II
S05M05	33%	10.6	−42.77	−281.38
	55%	10.2	−36.69	−119.04
	75%	10.8	−122.49	−398.45
S05M2	33%	30.4	−75.26	−457.30
	55%	47.9	−73.20	−894.68
	75%	30.9	−149.65	−375.48
S05M8	33%	5.9	−603.32	−2438.6
	55%			
	75%			
S08M05	33%	7.4	−56.50	−315.56
	55%	10.8	−53.03	−290.37
	75%	22.9	−97.42	−464.01
S08M2	33%	22.7	−38.11	−769.13
	55%	39.4	−69.13	−1088.0
	75%	64.9	−128.86	−943.98
S08M8	33%	39.9	−206.56	−2021.7
	55%	21.9	−143.19	−783.18
	75%			

**Table 6:** Fitting values for the creep deformation of sealed and partially dried S05M05 at 33, 55, and 75% relative humidity.

RH	*A* ($\frac{\mu \varepsilon }{\mathrm{MPa}}$)	*C* ($\frac{\mathrm{MPa}}{\mu \varepsilon }$)	τ (d)
Sealed	−5.978	−0.0337	216.471
75%	−37.7245	−0.00928	404.793
55%	−68.8956	−0.01068	130.265
33%	−42.1892	−0.01595	65.9869

## Data Availability

Not applicable.

## Abbreviations

The following abbreviations are used in this manuscript:

AAS	alkali-activated slag
OPC	Ordinary Portland Cement
s/b	solution-to-binder ratio
w/b	water-to-binder ratio
RH	relative humidity
UPV	Ultrasonic Pulse Velocity
